# Wearable Driver Distraction Identification On-The-Road via Continuous Decomposition of Galvanic Skin Responses

**DOI:** 10.3390/s18020503

**Published:** 2018-02-07

**Authors:** Omid Dehzangi, Vikas Rajendra, Mojtaba Taherisadr

**Affiliations:** Computer and Information Science Department, University of Michigan-Dearborn, Dearborn, MI 48128, USA; vrajendr@umich.edu (V.R.); mojtabat@umich.edu (M.T.)

**Keywords:** driver distraction, galvanic skin response, skin conductance, continuous decomposition analysis, spectro-temporal characterization, SVM-RFE feature selection

## Abstract

One of the main reasons for fatal accidents on the road is distracted driving. The continuous attention of an individual driver is a necessity for the task of driving. While driving, certain levels of distraction can cause drivers to lose their attention, which might lead to an accident. Thus, the number of accidents can be reduced by early detection of distraction. Many studies have been conducted to automatically detect driver distraction. Although camera-based techniques have been successfully employed to characterize driver distraction, the risk of privacy violation is high. On the other hand, physiological signals have shown to be a privacy preserving and reliable indicator of driver state, while the acquisition technology might be intrusive to drivers in practical implementation. In this study, we investigate a continuous measure of phasic Galvanic Skin Responses (GSR) using a wristband wearable to identify distraction of drivers during a driving experiment on-the-road. We first decompose the raw GSR signal into its phasic and tonic components using Continuous Decomposition Analysis (CDA), and then the continuous phasic component containing relevant characteristics of the skin conductance signals is investigated for further analysis. We generated a high resolution spectro-temporal transformation of the GSR signals for non-distracted and distracted (calling and texting) scenarios to visualize the associated behavior of the decomposed phasic GSR signal in correlation with distracted scenarios. According to the spectrogram observations, we extract relevant spectral and temporal features to capture the patterns associated with the distracted scenarios at the physiological level. We then performed feature selection using support vector machine recursive feature elimination (SVM-RFE) in order to: (1) generate a rank of the distinguishing features among the subject population, and (2) create a reduced feature subset toward more efficient distraction identification on the edge at the generalization phase. We employed support vector machine (SVM) to generate the 10-fold cross validation (10-CV) identification performance measures. Our experimental results demonstrated cross-validation accuracy of 94.81% using all the features and the accuracy of 93.01% using reduced feature space. The SVM-RFE selected set of features generated a marginal decrease in accuracy while reducing the redundancy in the input feature space toward shorter response time necessary for early notification of distracted state of the driver.

## 1. Introduction

The fatalities on the road have increased in 2016 by 5.6% percent from calendar year 2015 (37,461 lives were lost on U.S. roads in 2016) according to National Highway Traffic Safety Administration (NHTSA). The major contributing factor in the fatal accidents on the roadway is distracted driving. Continuous focus of the driver is a necessity for driving. Various research studies have shown that the driver’s attention decreases during multitasking, such as slower reaction time, decreased situational awareness, impairing judgments and narrowed visual scanning [1]. Distraction occurs when drivers divert their attention from the task of driving to a secondary activity instead such as having a phone conversation, texting, using the infotainment system [2], etc. The most common distracting secondary tasks during driving is when the driver uses his/her personal cell phone for either calling or texting. It is crucial to detect and notify driver distraction at its early stages in order to minimize the risk of road accidents. Many research investigations have been conducted to develop reliable feedback systems to alert distraction scenarios to the drivers. Many of the previous works employed techniques based on eye lid closure and movement tracking [3], lane tracking [4], and video cameras as an image processing technique by periodically taking video images of the driver [5] to identify inattention state of the drivers. Even though successful performances were achieved through above methods, they suffer from issues such as privacy violation risks and delayed detection and responses when the effect of distraction is visually noticeable. Those limitations can be overcome via continuous monitoring of physiological signals such as Electroencephalogram (EEG) rather than cameras. EEG based systems that generate state-of-the-art results are comprehensive and reliable [6] . However, the complexity of setup for collecting and analyzing the data is one of the major limitations of EEG, which makes the system expensive and intrusive to implement [7,8]. Galvanic Skin Response (GSR), on the other hand, is a minimally intrusive modality that can be sensed on the wrist and fingers and can be recorded easily [9,10]. GSR also known as skin conductance (SC) is one of the most sensitive markers for emotional arousal [11]. Unconscious response of our body to different stimuli through skin conductance is measured using GSR. Changes in skin conductance in the hands and foot region triggers emotional stimulation [11,12]. Higher skin conductance is demonstrated for intense level of arousal. Sympathetic activity, driving human behavior, cognitive, and emotional state on a subconscious level is controlled autonomously by the skin conductance.

Several investigations on synchronously recorded GSR signals have been conducted to inspect the impacts of cognitive state change. In study [12], the authors used GSR as an index of cognitive load to evaluate users’ stress due to workload while performing reading and arithmetic task. Temporal and spectral features were explored and concluded that spectral features showed to be promising in measuring the cognitive workload compared to the temporal features. In the previous work [13], a novel method for analyzing skin conductance (SC) using Short Time Fourier Transform (STFT) was employed to extract estimation of mental work load with high enough temporal bandwidth to be useful for augmented cognition application. Graphical data analysis of the STFT showed notable increase in the power spectrum across a range of frequencies directly following fault events. GSR was used in [14] for emotion recognition by extracting time domain and wavelet based features. Features were extracted using various window lengths. Random forest machine learning algorithm was used to characterize valence and arousal satisfactorily. In previous work [15], a system for human emotion recognition that automatically selects GSR features was proposed. Thirty features were extracted and a covariance based feature selection was implemented to extract an optimized feature set to better characterize the human emotions. Support vector machine (SVM) has been used for human emotion recognition with an accuracy of more than 66.67%. The above-mentioned previous works provided enough support to consider GSR as a reliable measure to identify and characterize mental workload. However, very few investigations were performed to detect cognitive workload or distraction while naturalistic driving using GSR. The authors in Ref. [16] used physiological signals like electrocardiogram, galvanic skin response and respiration to develop a novel system for stress detection during naturalistic driving. Features were extracted mainly from time, spectral and wavelet multi-domains. Features were generated for 10-s intervals of data. Detection of stress was accomplished using kernel-based classifiers. This study provided satisfactory results to employ physiological signal measures to in-vehicle intelligent systems to assist drivers on the road for early detection of stress. In that study, raw GSR signal in combination with other physiological measures was used to detect stress and solely time domain features of raw GSR were explored. In our previous work [10], we considered raw GSR signals for a preliminary analysis of driver distraction during a naturalistic driving experiment. We solely focused on two scenarios: (i) normal driving (non-distracted state) and (ii) driving while having an engaging phone conversation (distracted state). We then extracted some standard statistical measures and used binary SVM for distraction detection. Our aim was to analyze the discriminative power in the raw GSR space between normal and distracted driver state. We evaluated the detection model on six subjects and achieved the average detection accuracy of 91%.

Our aim in this paper is to design a system to identify the impact of secondary tasks of calling and texting on drivers using a continuous measure of phasic GSR signal during a naturalistic driving experiment. In our experiments, we use a wrist band wearable GSR on a population of 10 driver subjects that participated in this study during real driving experiments. Three scenarios were investigated in our experiments: (1) normal driving focusing attention on the primary task of driving; (2) phone distracted driving while having an engaging phone conversation; and (3) text distracted driving while writing and sending texts when driving. We hypothesize calling to be a cognitive distraction element in comparison to texting, which represent cognitive and visual distraction at the same time. We aim to evaluate GSR towards identification of distraction on the edge using short-term segments of GSR. The collected GSR data was decomposed into phasic and tonic components using continuous decomposition analysis (CDA) [17]. We then conducted a high resolution spectro-temporal analysis of the decomposed signals and continuous phasic components of GSR containing the most discriminative information was considered for subsequent analysis. We then extracted several spectral and temporal measures that characterize the phasic GSR signal in correlation with distracted scenarios. We employed linear and kernel-based Support Vector Machine (SVM) and 10 fold cross validation (10-CV) to generate identification results. Upon evaluating the result, phasic GSR showed promise as a reliable indicator of driver distraction by achieving an overall average accuracy of 94.81% to identify distraction elements under a naturalistic driving condition. Since input feature space is constructed in a manual process, the redundancy and computational complexity of the space might decrease the accuracy and response time of distraction identification in the generalization phase. Therefore, we employed support vector machine – recursive feature elimination (SVM-RFE) [18] to remove the redundancies for more efficient processing on the edge. We employed SVM-RFE in order for the following: (1) generate a rank of the discriminative features for the subject population, and (2) create a reduced feature subset with the highest distraction identification accuracy. Our experimental results using SVM-RFE demonstrated marginal decrease in accuracy while reducing the computational complexity and the redundancy in the input space towards early notification of distraction state to the driver.

## 2. Materials and Methods

**Figure 1:**
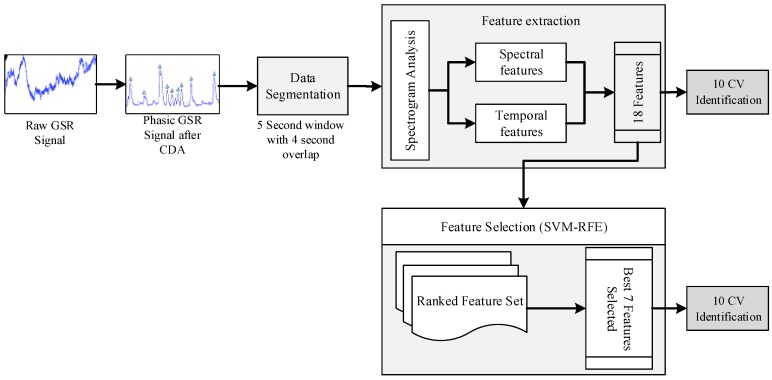
Flowchart of the proposed driver monitoring and intervention system on the edge. Galvanic Skin Responses (GSR); Continuous Decomposition Analysis (CDA); 10-fold Cross Validation (10-CV); Support Vector Machine Recursive Feature Elimination (SVM-RFE).

Figure 1 depicts a flowchart of the proposed driver monitoring and intervention system on the edge. After preprocessing, the recorded raw GSR signal was decomposed into phasic and tonic component using continuous decomposition analysis (CDA) and based on spectro-temporal analysis and characterization of the phasic GSR, we designed and developed segmentation and feature extraction modules. The extracted features were then used for identification tasks. Furthermore, feature selection was implemented using SVM-RFE to reduce the dimension of the feature space to alleviate the curse of dimensionality and improve the response time. In this section, we discuss the implementation of each step in detail.

### 2.1. Data Acquisition and Preprocessing

We have developed a custom designed wearable data acquisition platform comprising a synchronized multi-modal solution to acquire the physiological signals using a comprehensive wearable sensor network, which is used to collect data [19]. Our platform is capable of collecting large amount of heterogeneous drivers’ physiological including Galvanic Skin Response (GSR) during naturalistic driving.

Experiments conducted in this study were approved by the Institutional Review Board (IRB) of the University of Michigan with the Submission ID: HUM00102869. The driver participants were given a consent form to sign before the experiments, in which the nature of the data collection were described. The experiments were conducted under constant supervision of two research investigators for a reliable data acquisition process.

**Figure 2:**
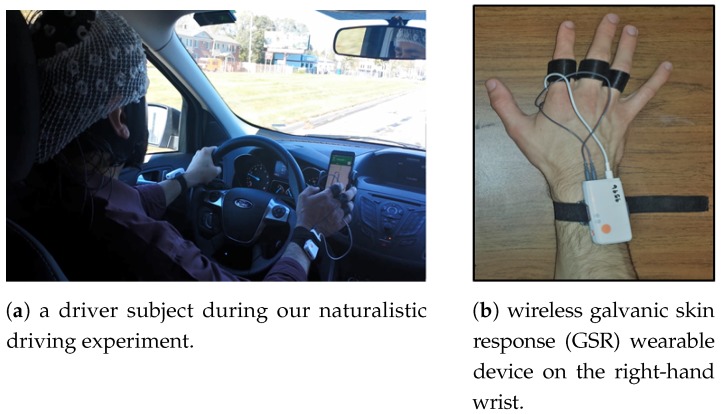
Experimental setup.

**Figure 3:**
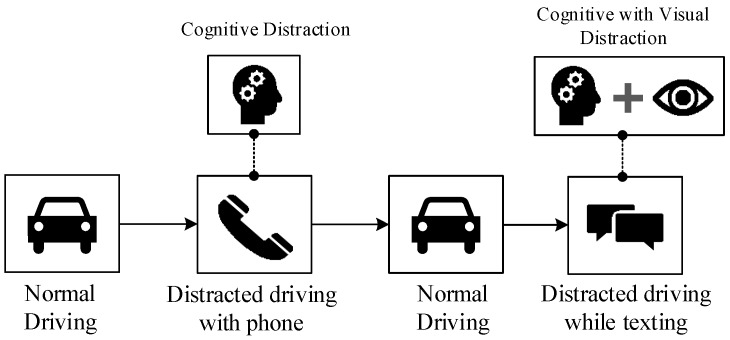
Illustrates data collection order during our naturalistic driving experiment.

The total of 10 subjects between the age group of 20–40 years that participated in our experiment were legally permitted to drive. Healthy male subjects were considered for our experiment to rule out the inconsistency. These subjects were told to stay away from any alcoholic beverages or pharmaceuticals that would trade off their sharpness amid the investigation. Figure 2a depicts a driver subject while conducting our naturalistic driving experiment. Three scenarios of driving was considered in our experiments, each of which were performed by the subjects for ≈2 min and their corresponding GSR signals were collected. The three driving scenarios were (i) driving under normal condition, (ii) driving while engaging in a phone conversation and (iii) driving while using the phone for texting. Normal driving (non-distracted) is represented by Scenario (i) while distracted driving is represented by scenarios (ii) and (iii). Figure 3 illustrates data collection order during our driving experiment. We employed a 10th order Butterworth low-pass filter <20 Hz on the recorded raw GSR to cope with and remove artifacts such as high frequency-noise, motion artifacts and also electromyography (EMG) artifacts that might interfere due to the movement of hand and finger during phone and texting experiment sessions.

### 2.2. Continuous Decomposition Analysis (CDA)

**Figure 4:**
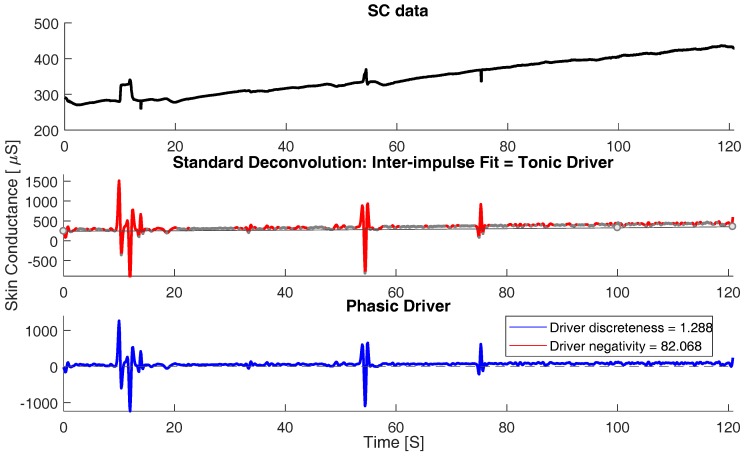
Continuous decomposition analysis (CDA) for normal scenario. (**top**): original skin conductance (SC) signal; (**middle**): decomposed Tonic and Phasic driver and (**bottom**): Phasic driver.

Skin conductance data is described by the superposition of subsequent skin conductance responses (SCRs). Due to this characteristic of the SCRs, the process of calculating the actual responses to a sympathetic activity in response to an external stimulus becomes tedious. This limitation is overcome by the deconvolution technique that separates the skin conductance (SC) data into phasic and tonic continuous activities. The tonic activity might include noise and shows subject dependencies. The tonic activity can be observed as a trend in the original SC signal in the top subplot of Figure 4. On the other hand, the phasic activity of the SC signal is considered for further investigation as this component of SC signal contains the actual response to any event-related sympathetic activity predominantly in the form of distinct burst of peaks with a zero baseline. The phasic component of SC also demonstrated trends in spectro-temporal space in correlation with the distracted scenarios.

Extracting the phasic component is done in three steps: deconvolution of galvanic skin response (GSR) data, computation of tonic activity and computation of phasic activity. A particular change in skin conductivity is triggered by secretion of sweat due to activity of the sudomotor nerve. In mathematical terms, the sudomotor nerve activity can be treated as a driver, containing distinct sequence impulse/bursts, which triggers a particular impulse response (i.e., SCRs). The outcome of this procedure can be described by convolution of the driver with impulse response function (IRF). IRF characterizes the shape of impulse response over time [17]:

(1) $$SC_{phasic}=Driver_{phasic}*IRF.$$

The phasic activity is believed to bestride a gradually changing tonic activity. Hence, SC activity can be considered to be composed as follows:

(2) $$SC=SC_{tonic}+SC_{phasic}=SC_{tonic}+Driver_{phasic}*IRF.$$

Tonic activity can also be considered as convolution of a driver function with same IRF. SC data can then be written as:

(3) $$SC=(Driver_{phasic}+Driver_{tonic})*IRF.$$

Deconvolution is the reverse process of convolution. Skin conductance data’s deconvolution incorporates a phasic and tonic fraction. By estimating one of them, the other can be determined easily:

(4) $$\frac{SC}{IRF}=Driver_{SC}=\left(Driver_{phasic}+Driver_{tonic}\right).$$

**Figure 5:**
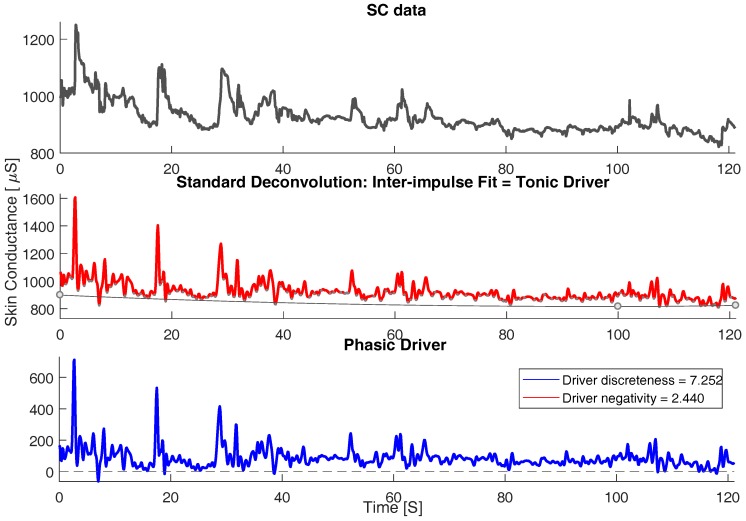
Continuous decomposition analysis (CDA) for distracted scenario, (**top**): original skin conductance (SC) signal; (**middle**): decomposed Tonic and Phasic driver and (**bottom**): Phasic driver.

Tonic electro-dermal activity can be observed in the absence of any phasic activity. However, SCRs (representing phasic SC activity) have a slowly recovering trail that may obscure any tonic SC activity. For the driver, the time constant of phasic responses is markedly reduced and so is their overlap. Time intervals between distinct phasic impulses can then be used to estimate tonic activity. Convolution can be conceived as a smoothing operation. Deconvolution has the reverse effect and amplifies error noise. Therefore, the resulting driver is smoothed by convolution with a Gaussian window. According to Equation (4), the phasic driver can now be computed by subtracting the tonic driver from the total driver signal. This subtraction results in a signal, which shows a virtually zero baseline and positive deflections reflecting the time-constrained nature of the phasic activity underlying the original SC data. The above methodology was driven from previous work [17]. Figure 4 and Figure 5 represents the deconvolution/decomposition of skin conductance signal for two scenarios, normal and distracted. The top most subplot in both the figures represents the original SC signal, the middle subplot represents deconvolution of tonic driver and the bottom subplot represents the phasic driver of the SC signal that contains the most discriminating component to characterize distraction. It can be observed from Figure 4 that, for a normal scenario, the phasic driver does not show impulse burst (bursts of consecutive peaks) as the subject is not under heavy workload. However, when we observe Figure 5 for the distracted scenario, more impulse bursts are observed as the subject is in a distracted state undergoing cognitive and visual distraction.

### 2.3. Spectral Analysis of Phasic Skin Conductance

**Figure 6:**
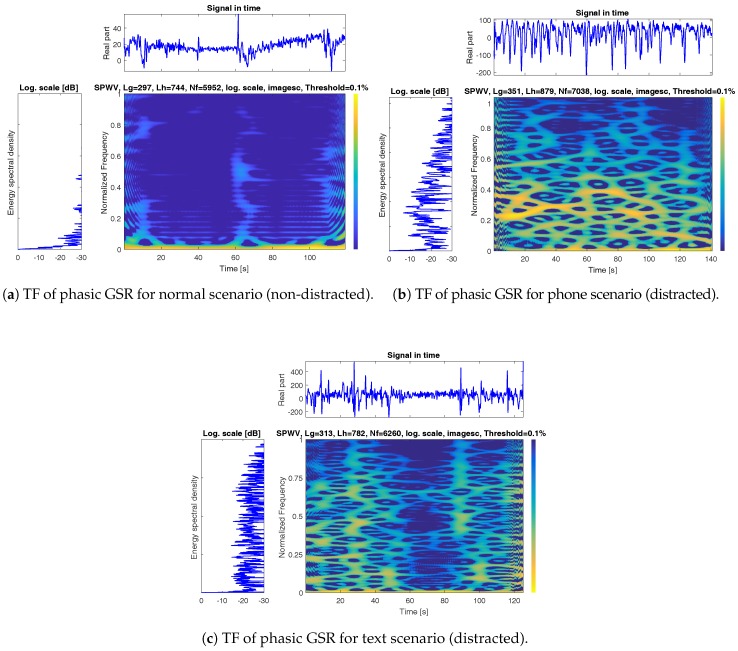
High-resolution time-frequency (TF) representation of the phasic component of GSR signal (**for each sub-plot**): (**top**): Phasic GSR Signal; (**left**): Energy Spectral Density and (**Spectrograph**): Time on the *x*-axis and frequency on the *y*-axis, and the color is used to indicate the power of the time-frequency sample.

Phasic SC as a non-stationary and multi-component signal varies in time and components over the time, and it needs to be analyzed in both temporal and spectral space to have a comprehensive evaluation on its characteristics. In order to investigate the spectro-temporal characteristics of the phasic SC signals related to different states of the driver, we conducted a Time-Frequency (TF) analysis and designed a high resolution TF representation. TF analysis could be considered as non-stationary signals analysis with frequency content varying with time. TF is a suitable representation for non-stationary and multi-component signals, which is able to describe the energy distribution of the given signal over time and frequency space simultaneously. The TF presents the beginning and end times of the different components of the signal as well as their frequency scope. To achieve the TF representation of the phasic SC signal, we use the Wigner–Ville Distribution (WVD) [20] approach attempting to attenuate the unwanted cross-terms in the TF space relative to the signal components. Figure 6 depicts TF representation of three scenarios, including phone, text, and normal states. Figure 6a–c represent normal, phone, and text scenarios, respectively, where the top panel in all of the sub-figures represents a raw phasic SC signal. The left panel depicts energy spectral density of a subject. The spectrograph represent time on the *x*-axis and normalized frequency on the *y*-axis, and the color is used to indicate the power of the TF sample. Based on the Nyquist frequency equation, with the sampling frequency of 50, the frequency range is 0–25 Hz, which was normalized to 0–1 Hz. The spectrograms were generated with normalized frequency observing the spectrograms in Figure 6, and we could identify considerably higher frequency components and peaks during distracted scenarios versus normal scenarios.

Based on our observations in the TF space, we designed a signal processing recipe for driver distraction identification on the edge for proactive monitoring and intervention. In the following sections, we will describe implementation of segmentation, feature extraction from spectral and temporal domain, feature selection using SVM-RFE and identification using linear and kernel-based SVM.

### 2.4. Segmentation and Window Analysis

To meet the requirement of short-response time for the proposed driver monitoring and intervention system on the edge, we implemented and employed a segmentation method to extract 5 s windows with 4 s overlap.

### 2.5. Feature Extraction

Several spectral and temporal features were extracted based on the analysis of the generated spectrograms from every window. The results of the calculated features were labeled accordingly and our feature space was generated using these sample data points. Extracted features shown in Table 1 are explained below:

* **Mean (1):** Sum of all the data points over total number of data points present in each window.
* **Variance (2):** Average of squared distance from mean.
* **Accumulated GSR (3):** Summation of GSR values in a window over total task time [11,12].
* **Maximum GSR value (4):** Maximum phasic GSR value in a window.
* **Power (5):** The sum of the absolute squares of a signals time-domain samples divided by the signal length, or, equivalently, the square of its root mean square (RMS) level.
* **Number of peaks based on second derivative (6):** The phasic GSR, P(i) signal’s first and second derivatives, $Q_{0}\left(i\right)$ and $Q_{1}\left(i\right)$ are calculated as in [21], as follows:
  (5) $$Q_{0}\left(i\right)=\left|P\left(i+1\right)-P\left(i-1\right)\right|,$$
  (6) $$Q_{1}\left(i\right)=\left|P\left(i+2\right)-2P\left(i\right)+P\left(i-2\right)\right|.$$
  These two arrays are scaled and then summed:
  (7) $$Q_{2}\left(i\right)=1.3*Q_{0}\left(i\right)+1.1*Q_{1}\left(i\right).$$
  This array is scanned until a threshold is met or exceeded:
  (8) $$Q_{2}\left(j\right)\geq 1.0.$$
* **Summation of amplitude of peaks (7):** The amplitude of number of peaks detected in a window previously is calculated and the amplitude is summed.
* **Short Time Fourier Transform (STFT) (8–11):** A technique quite often used to analyze physiological signal is STFT. Analysis of signals both in frequency and time domain can be performed using STFT. Fourier Transform is applied independently to each segment after dividing the original signal into equal segments. In [13], STFT analysis on SC data showed effective results in detecting work load. Based on our observation via spectro-temporal analysis of the phasic skin response, we found that most of the activity changes occur in the range of 0 to 50 Hz; therefore, we extracted four STFT coefficients each representing a sub-band with 12.5 Hz bandwidth between 0 and 50 Hz.
* **Fractal Dimensions (FD) (12–13):** A physiological signals chaotic or fractal nature is estimated using fractal dimension [22]. It is an impressive mathematical tool to model various complex physiological signals and an index to quantify the complexity of a fractal pattern. To characterize the time-series data, the fractal dimension technique is used generally. Fractal dimension was extracted using Higuchi and Katz methods.
* **Auto-Regressive (AR) (14–18):** The present output by an AR model of order p is calculated based on linear combination of past *p* output values along with some noise terms. By computing the weights on the previous *p* outputs of an auto-regression model, the mean squared error prediction can be minimized. The model with current output value y(n) and zero mean white space noise input x(n) is:
  (9) $$y\left(n\right)=\sum_{k=1}^{p}a\left(k\right)y\left(n-k\right)=x\left(n\right).$$
  In this study, the parameters of order *p* were selected as the features for each window. The order of *p* was set to 5, which generated five AR features.

### 2.6. Feature Selection Using Support Vector Machine—Recursive Feature Elimination (SVM-RFE)

SVM-RFE feature selection methodology based on support vector machine (SVM) was introduced by [23] and the authors used this methodology to select an important subset of features. It reduces the computational time for classification along with improvement of classification accuracy. The basis of SVM-RFE is backward removal of features iteratively. The main steps for feature selection is as follows [18]:

- Input the dataset to be classified,
- Weight of each feature is calculated,
- Removal of features having the smallest weight to obtain ranking of features.

Initially, entire features in the dataset is considered to compute ranking weights for all features. The linear SVM classifier is used to compute the rank weight of each feature. Then, iteratively, features with the lowest rank weight are discarded until only one feature remains in the dataset. Finally, the features will be listed in descending order of generated ranked weights. Algorithm 1 illustrates an iteration of SVM-RFE for ranking features in which the weight of each feature $\omega_{i}$ is given by

(10) $$\omega_{i}=\sum_{i}\left(\alpha_{i}y_{i}x_{i}\right),$$

where α is a linear SVM classifier used to train the given feature set *F* and is given by

(11) $$\alpha =SVM_{Train}\left(X,y\right),$$

and *X* is the input sample dataset and *y* is the corresponding class labels.
**Algorithm 1**: SVM-RFE for Ranking features **begin**  Given set of features, F⊂X  where X is the sample training dataset  Ranked set of features, R←∅   **repeat**   Train linear SVM with feature set *F*   Calculate the weight of each feature $\omega_{i}$    **For** each feature f∈F
**do**    Compute sorting standard: $c_{i}={\left(\omega_{i}\right)}^{2}$    **EndFor**   Find the feature with minimum weight, $f_{min}=arg_{min}${*c*}   Update $R=R\cup \left\{f_{min}\right\};F=F\backslash \left\{f_{min}\right\};$   **until** all features are ranked **end**: output *R*

### 2.7. Identification Task

To classify the original and transformed feature space, we employed linear and kernel-based support vector machine (SVM) with 10-fold cross validation. The SVM is a cutting edge discriminative learning model that aims to maximize the generalization capability of the predictor. SVM is a linear learner in nature, which can be boosted with nonlinearity using the kernel trick presented in [24]. SVM casts the input vector *x* into a scalar value g(x) as the output score,

(12) $$g\left(x\right)=\sum_{j=1}^{N}\alpha_{j}y_{j}K\left(x_{j},x\right)+c,$$

where the vectors $\left\{x_{j}|j=1,....,N\right\}$ are the support vectors, *N* is the number of support vectors, $\alpha_{j}>0$ are adjustable weights, $y_{j}=\left\{-1,+1\right\},c$ is the bias term, and the function $K(x_{j},x)$ is the kernel function. For the 2-class classification, the class decision is made based on the sign of g(x). As it can be seen, the classifier is constructed from sums of the kernel function expressed as,

(13) $$K\left(x_{j},x\right)=\omega {\left(x_{j}\right)}^{t}\omega \left(x\right),$$

where ω(x) is a mapping from the input space to a possibly infinite dimensional space. To model the nonlinear characteristics of the input data, kernel SVM functions such as Radial Basis function (RBF) and Polynomial function with two degrees of freedom (Poly d = 2) also known as quadratic kernel were employed. In order to extend the SVM to the multi-class task in hand, we employed one-vs.-one framework [24].

## 3. Results and Discussion

In this section, we provide the experimental analysis and results of the proposed methodology. In order to meet the short response time to identify distraction, the decomposed phasic GSR signal was segmented into 5 s windows with 4 s overlap and then extracted 18 different spectral and temporal features from each window based on our spectrogram observations. We employed linear and kernel-based SVM on the data corresponding to each subject separately for training and then evaluation of the predictive model using 10-fold cross validation (10-CV). In 10-CV, the original dataset is partitioned into 10 equal size subsets. Of the 10 subsets, a single subset is retained as the validation data for testing the model, and the remaining nine subsamples are used as training data. The cross-validation process is then repeated 10 times (the folds), with each of the 10 subsets used exactly once as the validation data. The 10 results from the folds can then be averaged to produce a single estimation. The advantage of this method is that all observations are used for validation in exactly one out of the 10 iterations without being participated in the training process whatsoever for that iteration.

### 3.1. Identification Results Using All the Features

Initially, the original 18D feature space was evaluated for identification generalization accuracy estimates. Table 2 reports the predictive model performance using all 18 features. Table 2 demonstrates that using all the 18 features the nonlinear polynomial d = 2 kernel SVM classifier achieved the highest average prediction accuracy of 94.81%, which shows that some critical discriminative information underlies in the nonlinear feature subspaces. It generated the results with an average prediction speed of 6620 observations per second and average training time of 0.86 s. From analyzing Table 3, the polynomial kernel classifier also demonstrated an average precision of 92.44% implying low false positive rate, recall of 96.38% implying even lower false negative rate, and f-score of 94.35%, the harmonic mean of the precision and recall, which are consistent with the accuracy results. It can be observed that linear SVM with an average accuracy of 91.94% was the fastest classifier achieving an average prediction speed of 7240 observations per second and an average training time of 0.75 s with a marginal decrease in average accuracy of 2.87%. This shows promising evidence that the generated feature space was effective in identifying the inattention state of the driver subjects even in the case of linear prediction.

### 3.2. Identification Results Using SVM-RFE Selected Feature Subset

To overcome the redundancy in the feature space, and computational complexity of the predictive learner due to the high dimensionality of the feature space, we employed SVM-RFE feature selection method. SVM-RFE also ranks the original feature space based on the feature correlation to the class label. It iterates through all the features calculating the correlation between the feature and class label and assigns a weight based on the level of correlation to the class label as described in Section 2.6.

Since the feature selection performed by SVM-RFE is a binary class technique, we considered two scenarios, namely (1) normal vs. phone and (2) normal vs. text. Scenario (1) was considered as cognitive distraction and scenario (2) was considered as cognitive and visual distraction. Table 4 shows the SVM-RFE ranking for normal vs. phone scenario and Table 5 shows the ranking for scenario normal vs. text. We observed a slight variance in the feature ranks between Table 4 and Table 5. However, considering subsets of features with various sizes, consistently similar set of features were selected for both distracted scenarios. In addition, by inspecting subject by subject responses in Table 4 and Table 5, a high level of similarity in the feature ranks across all subjects is observed. These observations helped us pick a unified set of features that are highly relevant for the task of distraction identification and are consistent between different scenarios and subjects. The best subset of features were selected by calculating the most frequently occurring features across all subjects. We then selected the first seven frequently occurring features as they demonstrated higher 10-CV identification accuracies compared to other feature subset sizes. We observed that, despite the fact that the order of first seven features is different for both scenarios, the set of the best seven features were the same for both distraction scenarios. The selected seven best features are listed below with their corresponding feature number as shown in subsection 2.5: (i) number of peaks in a window (6); (ii) STFT’s 2nd feature (9); (iii) Katz fractal dimension (13); and (iv–vii) Auto-regressive features (14, 15, 17 and 18).

Table 6 and Table 7 provide the identification results corresponding to the selected subset of 7D feature space in comparison with the results from 18D space. From Table 6 results using the subset of features that SVM-RFE selected, it is observed that we were able to achieve the average identification accuracy of 93.01% with a prediction speed of 7490 observations per second using polynomial kernel SVM classifier. When compared to the original 18D feature space, the results showed a marginal decrease of 1.8% in identification accuracy while providing a considerable improvement in the prediction speed (increase of 870 observations per second). From Table 7, we can see that the predictive model generated after reducing the feature space to 7D also demonstrated similar performance as the 18D predictive model for precision, recall and f-score measure of 89.61%, 95.83% and 92.50%, respectively. This minimal performance decrease in the identification accuracy from the original 18D feature space to 7D is a trade-off that results in improved response time for the online identification task in hand. Reducing the dimensions of the feature space can also potentially alleviate the effect of curse of dimensionality and increase the robustness of the predictive model in the generalization phase.

Table 6 demonstrates the nonlinear polynomial and RBF kernel SVM classifier generated the highest average accuracy of 93.01% and 91.61% respectively compared to linear SVM classifier with 88.35%. From Table 7, it can be observed that polynomial kernel SVM demonstrates the highest average precision, recall and f-score of 89.61%, 95.83% and 92.50%, respectively, which is consistent with the results based on the original feature space. These observations indicate that the selected subsets of features did not disrupt the discriminative capabilities of the original feature space by following similar performance trends to identify the distracted state from the non-distracted state with minimal decrease in the prediction accuracy and a considerable gain in response time.

## 4. Conclusions

In this study, we investigated if a continuous measure of phasic GSR can be used to identify distracted driving state under naturalistic driving conditions using a wrist band wearable. In contrast to other state-of-the-art driver monitoring and alerting systems using intrusive physiological signal measures such as EEG and ECG, GSRs are minimally intrusive. We evaluated GSR toward real-time identification of distraction using short-term segmented windows. We conducted decomposition on the raw GSR to obtain a continuous phasic GSR signal that contained the most discriminative characteristics to identify driver distraction. We then generated high resolution spectrograms of the phasic GSR signal to visualize and better understand its behavior. We extracted 18 features including spectral and temporal features that capture the pattern changes from normal to distracted scenarios at a physiological level. The nature of feature extraction is manual and might include redundancies that can increase the computational complexity and decrease the robustness of the predictor. Therefore, we employed the SVM-RFE technique to alleviate this limitation. SVM-RFE then selected seven features based on the rank assigned to the features that best characterized the distracted state from the non-distracted state. We employed linear and kernel-based SVM with 10-fold cross validation (10-CV) to generate identification results on both the original 18D and reduced 7D feature space. Further investigating the results demonstrated marginal reduction in prediction accuracy and considerable increase in the prediction prediction speed. The results across the population of subjects also demonstrated a high level of consistency. Our proposed driver monitoring and identification system on the edge provided evident results using GSR as a reliable indicator of driver distraction while meeting the requirement of early notification of distraction state to driver.

## Figures and Tables

**Table 1:** List of all the extracted spectral and spacial/temporal features.

Feature Domain	Feature Names
Spacial/temporal	Mean, Variance, Accumulated galvanic skin response (GSR), Average GSR, Maximum Value,
	Number of peaks and Sum of Amplitude of peaks,
	Fractal Dimensions, Auto Regressive
Spectral	Short Term Fourier Transforms (4 features)

**Table 2:** Identification results using linear and kernel-based (polynomial d = 2 and radial basis function) support vector machine (SVM) including accuracy, prediction speed, and training time with 18D feature space.

Subjects	Classifier Performance								
	Support Vector Machine								
	Linear SVM			Poly d = 2			Radial Basis Function (RBF)		
	Accuracy	Prediction Speed	Training	Accuracy	Prediction Speed	Training	Accuracy	Prediction Speed	Training
	in %	in obs/sec	Time in sec	in %	in obs/sec	Time in sec	in %	in obs/sec	Time in sec
Subject 1	82.90	6600.00	0.70	87.00	5700.00	0.74	85.40	5100.00	0.78
Subject 2	97.30	7700.00	0.75	97.30	7100.00	0.79	96.70	6800.00	0.81
Subject 3	92.30	6100.00	0.68	94.40	4900.00	0.77	90.60	4700.00	0.79
Subject 4	99.50	6100.00	0.74	99.20	5400.00	0.73	96.80	5300.00	0.79
Subject 5	87.10	6200.00	0.79	96.40	6400.00	1.37	87.40	6200.00	0.76
Subject 6	88.60	8000.00	0.74	93.60	7100.00	0.77	92.80	7300.00	0.89
Subject 7	98.00	6300.00	0.75	97.00	6300.00	0.73	97.50	6200.00	0.78
Subject 8	96.20	10000.00	0.75	97.40	8900.00	0.80	95.90	8500.00	0.90
Subject 9	84.00	8500.00	0.86	90.60	8000.00	1.08	87.40	7600.00	0.86
Subject 10	93.50	6900.00	0.75	95.20	6400.00	0.81	89.20	6300.00	0.81
**Average**	**91.94**	**7240.00**	**0.75**	**94.81**	**6620.00**	**0.86**	**91.97**	**6400.00**	**0.82**

**Table 3:** 10-fold cross validation (10-CV) identification results using linear and kernel-based (polynomial d = 2 and radial basis function) support vector machine (SVM) including accuracy, precision, recall and F-Score generated with 18D feature space.

Subjects	Performance Measures in %											
	Support Vector Machine											
	Linear SVM				Poly d = 2				Radial Basis Function (RBF)			
	Accuracy	Precision	Recall	F-Score	Accuracy	Precision	Recall	F-Score	Accuracy	Precision	Recall	F-Score
Subject 1	82.90	75.57	80.85	83.44	87.00	84.42	84.49	84.69	85.40	76.47	93.46	84.12
Subject 2	97.30	95.70	99.63	97.62	97.30	96.36	98.88	97.61	96.70	95.99	98.13	97.05
Subject 3	92.30	84.71	100.00	91.72	94.40	88.82	99.31	93.77	90.60	81.82	100.00	90.00
Subject 4	99.50	99.33	99.33	99.33	99.20	98.04	100.00	99.01	96.80	97.26	94.67	95.95
Subject 5	87.10	84.42	88.42	86.38	96.40	95.34	96.84	96.08	87.40	84.16	89.47	86.73
Subject 6	88.60	75.15	90.71	82.20	93.60	85.16	94.29	89.49	92.80	85.23	90.71	87.89
Subject 7	98.00	98.31	97.22	97.77	97.00	96.15	97.22	96.69	97.50	98.85	95.56	97.18
Subject 8	96.20	96.67	98.51	97.58	97.40	96.91	99.79	98.33	95.90	95.33	99.58	97.40
Subject 9	84.00	79.78	96.00	87.14	90.60	86.98	98.00	92.16	87.40	84.78	94.67	89.45
Subject 10	93.50	93.47	95.02	94.24	95.20	96.22	95.02	95.62	89.20	90.50	90.87	90.68
**Average**	**91.94**	**88.31**	**94.57**	**91.74**	**94.81**	**92.44**	**96.38**	**94.35**	**91.97**	**89.04**	**94.71**	**91.65**

**Table 4:** Support vector machine-recursive feature elimination (SVM-RFE) feature ranking for normal vs. phone.

Subjects	Normal vs. Phone																	
	RANK																	
	1	2	3	4	5	6	7	8	9	10	11	12	13	14	15	16	17	18
Subject 1	6	13	15	17	14	18	16	9	5	1	12	7	8	3	11	4	2	10
Subject 2	16	15	6	17	14	9	18	13	1	5	12	7	11	8	3	2	4	10
Subject 3	16	15	6	14	17	18	9	13	12	5	1	7	8	11	3	2	4	10
Subject 4	15	13	16	14	6	18	9	17	1	5	7	12	11	8	3	2	4	10
Subject 5	1	15	6	17	16	14	18	9	13	5	12	7	11	8	3	2	10	4
Subject 6	15	13	17	6	14	18	9	16	5	1	12	2	4	8	3	11	7	10
Subject 7	6	15	14	16	9	18	17	13	12	11	7	5	1	4	2	3	8	10
Subject 8	6	15	13	14	9	16	18	17	12	5	1	7	11	8	3	4	2	10
Subject 9	6	17	1	15	14	18	13	16	9	5	12	7	11	8	3	2	10	4
Subject 10	15	6	14	17	18	13	9	16	5	12	1	11	7	2	4	3	8	10
Frequent Feature	6	15	6	17	14	18	9	13	5	5	12	7	11	8	3	2	4	10

**Table 5:** Support vector machine-recursive feature elimination (SVM-RFE) feature ranking for normal vs. text.

Subjects	Normal vs. Text																	
	RANK																	
	1	2	3	4	5	6	7	8	9	10	11	12	13	14	15	16	17	18
Subject 1	15	6	17	14	18	9	16	13	5	1	12	7	11	3	8	2	10	4
Subject 2	15	6	17	14	18	16	9	13	12	7	11	5	1	3	8	10	2	4
Subject 3	5	6	15	17	18	14	16	9	13	1	12	7	11	3	4	2	8	10
Subject 4	17	15	13	18	14	6	16	9	1	12	5	7	11	3	8	2	10	4
Subject 5	6	17	15	16	14	18	9	13	5	1	12	7	11	3	8	2	4	10
Subject 6	17	16	6	15	18	14	9	13	1	2	4	12	5	8	3	11	7	10
Subject 7	15	17	9	14	18	16	13	12	8	11	10	7	6	5	4	3	2	1
Subject 8	5	15	6	17	14	18	16	9	13	12	7	8	11	10	4	3	2	1
Subject 9	5	6	17	15	18	14	9	16	13	1	12	7	11	8	3	2	4	10
Subject 10	15	17	12	14	18	13	9	6	16	11	5	1	8	3	2	4	7	10
Frequent Feature	15	6	17	14	18	14	9	13	13	1	12	7	11	3	8	2	2	10

**Table 6:** Identification results of linear and kernel-based (polynomial d = 2 and radial basis function) support vector machine (SVM) including accuracy, prediction speed, and training time with the reduced 7D Feature Space.

Subjects	Classifier Performance								
	Support Vector Machine								
	Linear SVM			Poly d = 2			Radial Basis Function (RBF)		
	Accuracy	Prediction Speed	Training	Accuracy	Prediction Speed	Training	Accuracy	Prediction Speed	Training
	in %	in obs/sec	Time in sec	in %	in obs/sec	Time in sec	in %	in obs/sec	Time in sec
Subject 1	79.70	7000.00	0.68	85.60	6200.00	1.42	84.60	6300.00	0.69
Subject 2	97.10	9200.00	0.68	96.30	8600.00	1.89	97.30	8000.00	0.76
Subject 3	85.80	6600.00	0.75	91.20	5700.00	1.39	88.50	5800.00	0.71
Subject 4	99.70	7300.00	0.66	99.70	5700.00	0.79	98.70	5000.00	0.77
Subject 5	75.00	7800.00	1.27	95.40	7300.00	3.70	88.80	6500.00	0.78
Subject 6	82.90	8800.00	0.75	88.60	8700.00	0.76	86.80	8000.00	0.70
Subject 7	94.00	7900.00	0.65	96.30	7100.00	0.69	95.80	5500.00	0.78
Subject 8	92.50	12000.00	0.78	93.80	10000.00	0.77	94.60	9600.00	0.89
Subject 9	81.40	9800.00	1.49	88.00	8400.00	1.13	86.50	8200.00	0.77
Subject 10	95.40	8200.00	0.66	95.20	7200.00	0.77	94.50	7000.00	0.70
**Average**	**88.35**	**8460.00**	**0.84**	**93.01**	**7490.00**	**1.33**	**91.61**	**6990.00**	**0.76**

**Table 7:** 10-fold cross validation (10-CV) identification results using linear and kernel-based (polynomial d = 2 and radial basis function) support vector machine (SVM) including accuracy, precision, recall and F-Score with the reduced 7D feature space.

Subjects	Performance Measures in %											
	Support Vector Machine											
	Linear SVM				Poly d = 2				Radial Basis Function (RBF)			
	Accuracy	Precision	Recall	F-Score	Accuracy	Precision	Recall	F-Score	Accuracy	Precision	Recall	F-Score
Subject 1	79.70	72.94	81.05	76.78	85.60	80.12	86.93	83.39	84.60	75.26	93.46	83.38
Subject 2	97.10	95.36	99.63	97.45	96.30	95.29	98.13	96.69	97.30	95.70	99.63	97.62
Subject 3	85.80	75.26	99.31	85.63	91.20	83.14	99.31	90.51	88.50	78.69	100.00	88.07
Subject 4	99.70	99.34	100.00	99.67	99.70	99.34	100.00	99.67	98.70	98.01	98.67	98.34
Subject 5	75.00	69.16	82.63	75.30	95.40	93.85	96.32	95.06	88.80	83.33	94.74	88.67
Subject 6	82.90	65.41	86.43	74.46	88.60	75.76	89.29	81.97	86.80	75.00	81.43	78.08
Subject 7	94.00	96.43	90.00	93.10	96.30	96.09	95.56	95.82	95.80	95.53	95.00	95.26
Subject 8	92.50	92.93	97.66	95.24	93.80	93.39	98.94	96.08	94.60	93.98	99.36	96.59
Subject 9	81.40	76.94	95.67	85.29	88.00	83.33	98.33	90.21	86.50	81.49	98.33	89.12
Subject 10	95.40	96.62	95.02	95.82	95.20	95.83	95.44	95.63	94.50	94.29	95.85	95.06
**Average**	**88.35**	**84.04**	**92.74**	**87.87**	**93.01**	**89.61**	**95.83**	**92.50**	**91.61**	**87.13**	**95.65**	**91.02**
